# Allosteric effects of CDR-H3 mutations modulate binding and neutralization of a conserved SARS-CoV-2 RBD-targeting antibody

**DOI:** 10.1128/spectrum.04127-25

**Published:** 2026-08-05

**Authors:** Muhammad Waqas Nasir, Qiyun Liang, Jun He, Umer Rashid, Jiabing Wu, Yong Gao

**Affiliations:** 1 The First Affiliated Hospital of USTC, Division of Life Sciences and Medicine, University of Science and Technology of China 12652, Hefei, Anhui, China; 2 Anhui Provincial Center for Disease Control and Prevention 117844, Hefei, China; 3 Hefei Infectious Disease Hospital 714924, Hefei, China; 4 Department of Chemistry, COMSATS University, Islamabad, Abbottabad, Pakistan; Northwest A&F University College of Animal Science and Technology, Yangling, Shaanxi, China

**Keywords:** SARS-CoV-2, spike receptor-binding domain (RBD), monoclonal antibody XG83, CDR-H3, alanine scanning, antibody engineering, Omicron variant, molecular dynamics

## Abstract

SARS-CoV-2 antigenic evolution continues to erode the activity of first-generation monoclonal antibodies, underscoring the value of antibodies that recognize conserved features within the spike receptor-binding domain (RBD). As a model for breadth-oriented engineering, we assessed the RBD-directed antibody XG83, which has a CDRH3-dominated paratope. Utilizing the XG83-Wuhan RBD crystal structure as a reference, we integrated MOE alanine scanning and residue scanning with CDRH3 (A116-Y132) to identify chemically reasonable replacements and test interaction to an Omicron BA.1 RBD. The parental CDRH3-centric pose in comparative docking had a better score (−280.18) than the mutant MuXG83 (−254.2), along with 100-ns molecular dynamics showed that MuXG83 was less stable (with higher RMSD/RMSF with less favorable interaction energy). The ELISA results against Omicron BA.1 RBD demonstrated that XG83 had 40% stronger binding than MuXG83; convergence happened only at the highest concentration, concordant with SPR studies and the fact that mutation increased dissociation. According to this binding gap, BA.1 pseudovirus neutralization demonstrated that XG83 was much more powerful than MuXG83, showing that the E118L/F130H CDR-H3 alterations decreased functional activity against Omicron BA.1. These results imply that allosteric influences on interface stability and conformational dynamics by non-epitope CDR-H3 residues can affect antibody performance. Functional testing was confined to Omicron BA.1; therefore, larger variant-panel studies are needed to ascertain if such mutations affect antibody breadth. Our findings highlight a structure-guided approach for optimizing paratopes and indicate that non-epitope (potentially allosteric) changes to CDRH3 are also important while investigating potential development and neutralization before advancement.

**IMPORTANCE:**

This study shows how modest allosteric characteristics in CDR-H3 control the delicate balance between neutralizing potency and breadth, making a timely and significant addition to SARS-CoV-2 antibody engineering. Using crystallography, alanine scanning, residue scanning, docking, molecular dynamics, and experimental ELISA and neutralization assays, this study offers a structure-guided framework for rational paratope optimization. The discovery of CDR-H3 residues that regulate long-range stability rather than just direct epitope contacts reveals an unappreciated aspect of antibody design and explains why some alterations improve anticipated interactions but degrade functional performance. Importantly, the comparison of wild-type XG83 and modified MuXG83 shows how allosteric tuning affects antibody-antigen compatibility in developing variations like Omicron. These findings illuminate conserved RBD recognition and offer strategies for building next-generation therapeutic antibodies that are more resistant to viral evolution.

## INTRODUCTION

SARS-CoV-2, the causative agent of COVID-19, catalyzed a global health crisis beginning in the late 2019. Among the most effective immune defenses against this virus are neutralizing antibodies (nAbs) that target the spike glycoprotein, particularly its receptor-binding domain (RBD), to block attachment to the human ACE2 receptor (1, 2). Yet, the virus continues to diversify, and recurrent mutations in spikes challenge first-generation antibodies, sharpening the focus on antibodies that recognize structurally conserved epitopes. Accordingly, the field’s focus has shifted toward neutralizing antibodies that recognize conserved epitopes on the RBD, which are more likely to retain activity as the virus evolves (3).

While numerous SARS-CoV-2 nAbs have been discovered and structurally characterized, the determinants that enable broad and durable neutralization remain incompletely understood. RBM-directed antibodies (class 1/2) achieve exceptional potency by mimicking ACE2 contacts but often lose effectiveness when even a single amino acid changes within that region (4, 5). In contrast, class 3/4 antibodies that target conserved, non-RBM regions of the RBD tend to retain breadth but may sacrifice some potency. Understanding how these antibodies achieve resilience, what structural features and energetic interactions permit sustained recognition across variants remains a central challenge (6).

This study centers on the monoclonal antibody XG83, a human nAb raised against the SARS-CoV-2 RBD. XG83 is compelling as a model because it appears to engage a conserved surface inside the receptor-binding motif (RBM) while maintaining strong heavy-chain CDR-H3 interactions (7). The core hypothesis is defining the energetic and structural contributions of XG83’s paratope and epitope including both direct contacts and allosteric effects would reveal general principles for antibody breadth and stability. Specifically, the study proposed that targeted CDR-H3 modifications may alter XG83 binding by reshaping local packing and long-range paratope dynamics. Rather than assuming improved breadth, this study evaluates whether selected non-epitope CDR-H3 substitutions preserve or disrupt binding and neutralization against Omicron BA.1. However, to test this hypothesis, we combined complementary computational and structural approaches. The analysis of the crystal structure of XG83 bound to the wild-type RBD, modeled the XG83 Omicron complex to assess variant compatibility, and employed molecular dynamics simulations to characterize conformational flexibility and stability. In parallel, alanine-scanning mutagenesis both computational and experimental was used to quantify the energetic importance of individual residues on the paratope and epitope (8, 9). Together, these methods identified key contact residues within CDR-H3 and conserved hot spots on the RBD that anchor recognition. The results suggest that even subtle framework or second-shell changes can propagate through local networks to modulate affinity and stability.

Taken together, these findings establish a mechanistic framework for conserved RBD recognition and identifying structural levers such as CDR-H3 orientation and hotspot conservation that can be exploited to enhance antibody breadth (10). This integrative approach clarifies how XG83 engages its target and provides general insights for the rational engineering of next-generation antibody therapeutics resilient to viral evolution.

## MATERIALS AND METHODS

### Antibody source and crystallization, data collection, refinement, and analysis

Human monoclonal antibody XG83 was produced recombinantly from published heavy- and light-chain sequences originally isolated from convalescent COVID-19 donors; no new human subjects were recruited. The SARS-CoV-2 antigens comprised Wuhan-Hu-1 and Omicron RBDs (spike residues 319–541), codon-optimized, His-tagged, and cloned into pTT5 for transient expression in HEK293F cells. Following 6-day expression, RBDs were purified by Ni-NTA and quality-checked by SDS-PAGE. XG83 variable regions were synthesized and cloned into human IgG1 expression backbones (VRC01-derived); heavy and light chains were co-transfected into HEK293F (PEI; 2 × 10^6^ cells/mL), and IgG was purified by Protein A, concentrated with 10-kDa MWCO units, quantified spectrophotometrically, and stored at −80°C. For structure determination, XG83 IgG was digested with papain (1:50, enzyme: antibody, 1 h) to generate Fab. The reaction was quenched and passed over Protein A to retain Fc, collecting Fab in the flow-through; Fab and Wuhan RBD were further polished and concentrated and then mixed on ice for 1 h. The Fab-RBD complex was isolated by size-exclusion chromatography (HiLoad 16/600 Superdex 200 pg; 20 mM Tris pH 8.0, 0.15 M NaCl; 1 mL/min), pooling the complex peak after SDS-PAGE verification. Crystallization used commercial screens followed by optimization, yielding best diffraction with 0.15 M lithium sulfate monohydrate, 0.1 M citric acid (pH 3.5), and 18% PEG 6000; crystals were cryoprotected with 25% ethylene glycol. Data were collected at SSRF BL02U1 to 2.95 Å, processed with XDS/AIMLESS, solved by molecular replacement in PHASER (template PDB 7MU4), and iteratively built/refined in COOT/PHENIX to final R work/R free of 25.2%/29.5%. The refined model has been deposited in the PDB under accession 9UTF.

### Structural analysis, modeling, and docking

The wildtype XG83-Wuhan RBD crystal structure XG83 Fab: PDB 9UTF (https://www.rcsb.org/structure/9UTF) was inspected in MOE (2016.0802) and PyMOL for interface definition and model preparation. For Omicron, the RBD (PDB 8KHD) and the XG83 Fab were prepared and docked using HDOCK and ClusPro with default/global settings and no restraints (Fab as receptor; RBD as ligand). For each server, the top 10 solutions were ranked by cluster size and scoring energy; the centroid of the largest, best-scoring cluster from ClusPro was selected and cross-validated against HDOCK for interface consistency (11, 12).

### Alanine scanning and mutational modeling

To identify critical residue contacts at the XG83-RBD interface, an *in-silico* alanine scanning mutagenesis was performed. We focused on the XG83 paratope, particularly residues in the complementarity-determining region H-3 (CDRH-3) that directly contact the RBDs. Using Molecular Operating Environment (MOE 2016, Chemical Computing Group) software, each targeted residue on XG83 was systematically mutated to alanine *in silico*, and the effect on binding energy and stability was evaluated (13). The XG83-Omicron RBD complex structure (from docking) was input into the MOE Protein Design tool, and an alanine scan was run for all residues in the CDRH-3. MOE’s scoring function computed the change in binding affinity (ΔΔG, reported as “ΔAffinity”) upon each Ala substitution (7). A positive ΔAffinity indicates that the mutation weakened binding (i.e., the wild-type residue contributed favorably to binding), whereas a negative ΔAffinity suggests the mutation improved binding (the wild-type residue was unfavorable). The ΔΔG thresholds were applied as operational screening criteria for mutation prioritization rather than as absolute biological cutoffs; substitutions with ΔΔ*G* ≥ +2.0 kcal/mol were classified as strongly deleterious putative hotspot residues, whereas substitutions with ΔΔ*G* ≤ −1.0 kcal/mol were considered potentially favorable candidates for further residue scanning and experimental validation. The alanine scanning identified a subset of positions in XG83’s heavy-chain CDR3 (HCDR3), spanning residues 117–132 (IMGT numbering), that were particularly important. These computational alanine scan results highlighted “hotspot” residues at the interface that contribute most to binding free energy.

### Residue scanning and *in silico* mutagenesis

The XG83-Omicron complex was subjected to alanine scanning followed by comprehensive residue scanning to assess the contribution of individual amino acids to binding affinity and structural stability. In this approach, each residue is systematically substituted with the remaining 19 amino acids, and potential steric clashes are evaluated. This method, widely used in antibody engineering, drug development, and epitope mapping, identified positions E118 and F130 as promising mutation sites due to their moderate impact on binding and no contribution in interaction in all docking poses. Substitutions with leucine and histidine, respectively, were selected to check the impact of these on stability and binding. Mutant antibody models were generated in PyMOL using the mutagenesis wizard, which allowed sidechain rotamers to be adjusted to minimize clashes. Each XG83 variant was subsequently refined by local energy minimization in Gromacs with the RBD present to relieve substitution-induced strain.

To evaluate the binding potential of the variants, computational affinity estimation was performed using both MOE’s built-in affinity scoring and Discovery Studio’s Calculate Binding Energy protocol, the latter employing a physics-based forcefield with implicit solvent. The change in binding free energy (ΔΔ*G*-mut) relative to the wild-type complex was calculated, and only variants consistently predicted by both methods to significantly improve binding (ΔΔ*G*-mut < −0.5 kcal/mol, indicative of tighter association) were retained. These promising variants were designated MuXG83 antibody. Docking, molecular dynamics simulations, and interface analyses were then repeated for the mutant complexes to confirm the preservation of key contacts and geometric complementarity. Through this iterative workflow, several single-residue MuXG83 variants were identified that demonstrated improved predicted binding energies while maintaining structural integrity at the antibody-RBD interface.

### Molecular dynamics simulations

To probe complex stability and motions, MD simulation was imposed through GROMACS 2024.4: (i) wild-type XG83 Fab bound to Omicron RBD (Docked Complex) and (ii) Mutated XG83 (MuXG83 Fab bound to Omicron RBD (top-ranked docked model). Each complex was placed in a triclinic box with ≥1.0 nm padding under periodic boundaries, solvated with TIP3P water, neutralized, and brought to 0.15 M NaCl. After solvation, structures were energy-minimized by steepest descent until the maximum force was <1,000 kJ·mol⁻¹·nm⁻¹. Equilibration proceeded in two stages with protein heavy-atom restraints: NVT at 300 K for 100 ps (V-rescale thermostat), followed by NPT at 300 K and 1 bar for 100 ps (Parrinello-Rahman barostat) to stabilize pressure and achieve ~1 g mL⁻¹ water density (14–16). Production trajectories were then run for 100 ns; the initial 1–2 ns were discarded as additional equilibration. Analyses used standard GROMACS tools and custom Python scripts: Cα-fitted RMSD (gmx rms) for global drift, per-residue RMSF (gmx rmsf) for flexibility, radius of gyration (gmx gyrate) for compactness, SASA (gmx sasa) for exposure changes, and intermolecular H-bonds (gmx hbond) to monitor interface persistence. Principal component analysis was performed on covariance matrices to characterize dominant collective motions over the equilibrated production windows.

### Enzyme-linked immunosorbent assay

A 200 ng/well coating of stabilized soluble SARS-CoV-2 Omicron spike protein RBD in 1× PBS was applied to Nunc Maxisorp ELISA plates (ThermoFisher) and left at 4℃ for 16 h. The coated wells were then incubated at 37℃ for 2–3 h with 200 μL of blocking buffer, which is PBS with 5% BSA. Afterward, 300 μL of washing buffer, which is PBS with 0.1% Tween 20, was used to wash the wells three times. The SARS-COV-2 spike protein RBD-coated wells were incubated in a twofold series of dilutions of the SARS-COV2 antibodies for 1 h at 37℃. PBS was utilized as a blank control. Following three washes with washing buffer, the wells were incubated for 1 h at 37℃ with a 1:250 dilution of goat anti-human IgG secondary antibody. After adding the TMB (Beyotime) substrate and letting it react in the dark for 15 min, we added the Stop Solution to stop the color development. An ELISA Analyzer 8 with a Touch Screen MB-580 was used to detect absorbance at 450 nm. The ELISA experiments were performed in three independent biological replicates, with each antibody concentration measured in technical triplicate wells. OD450 values were blank-subtracted using PBS-only control wells and reported as mean ± SD. Binding curves were generated by plotting OD450 against antibody concentration.

### Pseudo-virus neutralization

This section details the procedures used to conduct a neutralization assay with SARS-CoV-2 pseudovirus in order to determine whether XG83 and MuXG83 were effective in blocking Omicron variant entry. 96-well plates were seeded with 10,000 cells/well of HEK293T/hACE2 cells in culture media 24 h prior to the test. After being fivefold serially diluted in culture medium, the testing antibodies were incubated with 50 μL of pseudovirus at 37°C for 1 h. The initial dilution for antibody dilution was 1 mg/mL. After that, HEK293T/hACE2 cells were treated with the antibodies and virus mixture and left to incubate at 37°C with 5% CO2 for an additional 48 h. Lastly, the Britelite plus Reporter Gene Assay System (PerkinElmer) was used to evaluate the luciferase activity of HEK293T/hACE2 cells infected with Pseudovirus. Utilizing fitting nonlinear regression curves in GraphPad Prism 10.4.1, we were able to calculate the highest antibody dilution that resulted in 50% pseudovirus neutralization (NTIC50).

### Surface plasmon resonance assay

Surface plasmon resonance (SPR) analysis was performed using a Biacore 8K instrument (Cytiva) equipped with a CM5 sensor chip. Omicron BA.1 RBD was immobilized on the sensor surface by amine-coupling chemistry in 10 mM sodium acetate buffer, pH 4.0. SPR measurements were conducted in PBS containing 0.005% Tween-20 at a flow rate of 30 μL/min. WT XG83 and MuXG83 antibodies were injected over the immobilized RBD surface at five serial concentrations. Each injection included a 120-s association phase followed by an 1,800-s dissociation phase. The sensor surface was regenerated with 20 mM NaOH after each cycle. Binding kinetics were analyzed using a 1:1 Langmuir binding model in Biacore Insight Evaluation Software v3.0.

### Statistical analysis

By comparing the RLU values with the negative control wells, the OD450 and neutralization were determined using GraphPad Prism 10 (GraphPad Software) as the dilution at which the RLU values were reduced. GraphPad Prism 10 was used to examine the statistical significance of the difference in OD450. A non-linear regression analysis was conducted to examine the relationship between anti-SARS-CoV-2 antibodies and Omicron RBD and determined the binding affinity and neutralization by plotting the curves.

## RESULTS

### Structural and confirmational analysis of CDRH3 in XG83 complex

**Fig 1:**
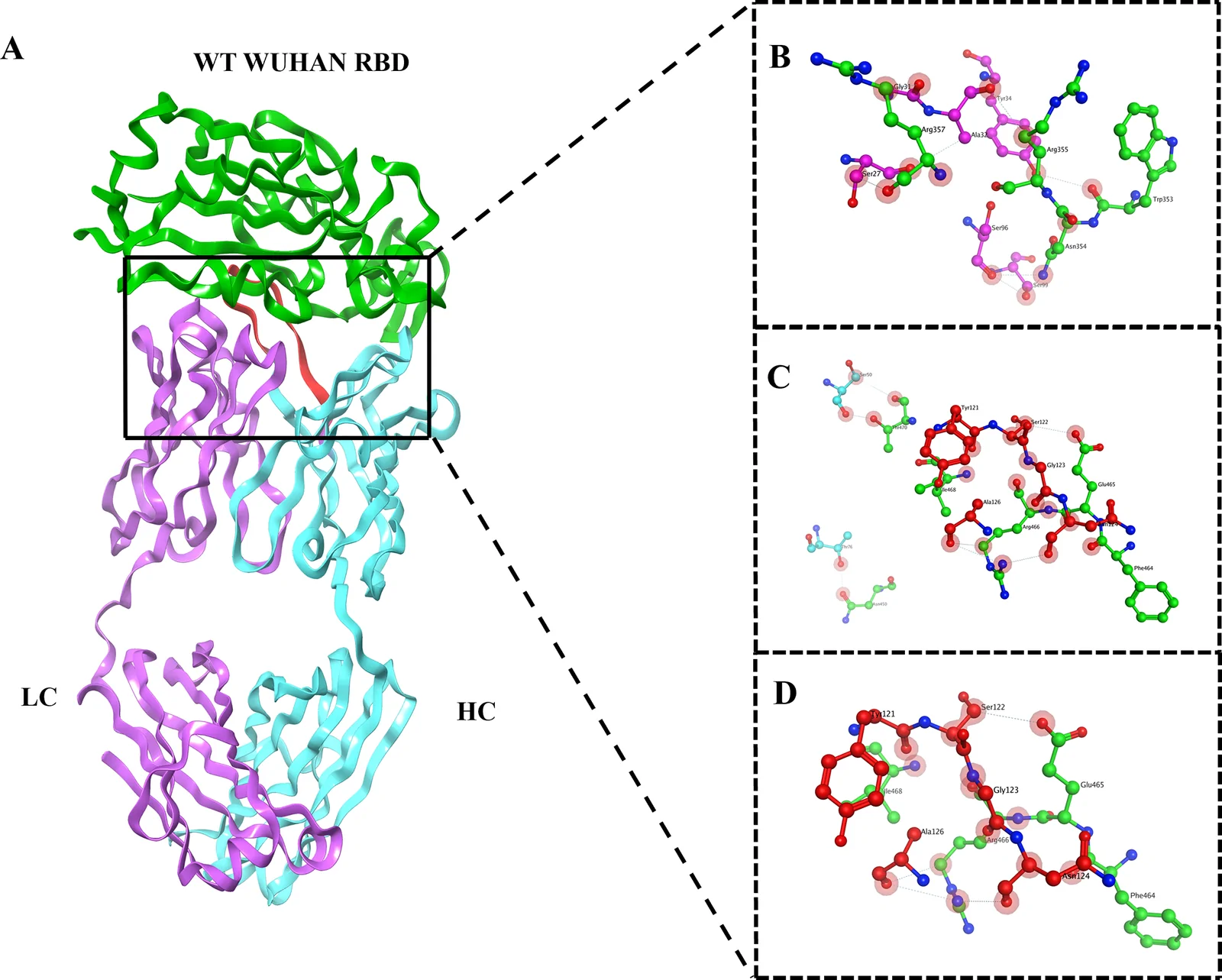
Structural architecture of the XG83 Fab in complex with the Wuhan wild-type RBD, highlighting the CDRH3-dominated interface. (**A**) Overall crystal structure of the XG83 Fab bound to the Wuhan RBD. The variable heavy (HC) domain is shown in cyan, the variable light (LC) domain in magenta, and the RBD in green. The CDRH3 loop of XG83, the principal determinant of antigen recognition, is colored red. The Fab-RBD interface forms a compact paratope-epitope network characterized by extensive hydrogen bonding and hydrophobic packing. (**B**) Interface view showing light-chain CDRs (magenta) complementing the heavy-chain paratope to stabilize the antigen-antibody complex. The cooperative network of hydrogen bonds and solvent bridges reinforces the structural integrity of the Fab-RBD interaction. (**C**) Close-up view of the CDRH3 loop (red) engaging the Wuhan RBD. Key aromatic and charged residues (Tyr128, Tyr129, Phe130, Arg117, Asp131) participate in π-π stacking, hydrogen bonding, and water-mediated contacts (red spheres), anchoring the loop within the RBD groove. This configuration underscores the dominance of CDRH3 in shaping binding specificity and affinity. (**D**) Residual interaction between Wuhan RBD and CDRH3 regions.

The XG83 Fab-Wuhan RBD complex adopts a well-defined binding geometry in which the variable domains of the heavy (HC, Cyan) and light (LC, magenta) chains form a contiguous antigen-binding surface. The RBD (green) interfaces primarily with the heavy-chain paratope, positioning the complementarity-determining region H3 (CDRH-3, red) at the center of the binding cleft (Fig. 1A). The overall conformation reveals a compact interface stabilized by multiple hydrogen-bonding and hydrophobic interactions. In the Wuhan wild-type RBD, the XG83 CDRH-3 loop (Ala116-Tyr132) adopts an extended β-hairpin that inserts deeply into a recessed groove of the RBD. Aromatic residues Tyr128, Tyr129, and Phe130 form the core of the interface, engaging in π-π stacking and van der Waals contacts with hydrophobic side chains on the RBD. Polar residues Arg117 and Asp131 complete a hydrogen-bonding network with neighboring RBD residues, with several interactions mediated by ordered water molecules. This combination of hydrophobic and polar contacts highlights CDRH-3 as the principal contributor to antigen recognition and binding affinity. Although the heavy chain dominates the interaction, the light-chain CDRs, particularly CDR-L3, complement the interface by stabilizing the heavy-chain approach (Fig. 1B and C). Key contacts involve side-chain hydrogen bonds and water-mediated interactions that assist in orienting the paratope for optimal engagement. The interface shows a cooperative packing arrangement, supporting the stability of the Fab-RBD complex.

Compared with unbound models, CDRH3 exhibits moderate conformational flexibility, reflected in small deviations in backbone dihedral angles and side-chain orientations, suggesting induced fit upon antigen binding. This dynamic adaptability is consistent with the chemical heterogeneity of CDRH3 and supports its central role in antigen recognition (Fig. 1D). The X-ray crystal structure of the XG83 Fab in complex with the Wuhan wild-type RBD was refined to a resolution of 2.95 Å, which is well within the acceptable range for high-confidence macromolecular models (reference threshold <3.10 Å). The data set exhibited excellent completeness (95.63%; 97.89% in the highest resolution shell), ensuring robust coverage of reciprocal space. The CC1/2 value of 0.995 (0.775 in the highest shell) indicates strong internal data consistency and high-quality diffraction. The value of 0.2209 (1.769 in the highest shell) further supports reliable measurement precision. Model geometry and stereochemical quality were validated using MolProbity, yielding a MolProbity score of 2.13, which falls within the expected range for well-refined antibody-antigen complexes (<2.20). The Ramachandran plot shows 96.42% of residues in favored regions and no outliers, confirming accurate backbone conformations and minimal model strain. A clash score of 6.35 indicates few steric clashes between nonbonded atoms, reflecting the model’s structural reliability. In total, 627 protein residues were built into the final model, encompassing both Fab chains and RBD (Table S1).

### Omicron RBD-XG83 complex: conserved CDR-H3 core, allosteric residue effects, and structural impact of residue scanning

In docking XG83 to the Omicron RBD, it yields a single, well-packed pose in which the paratope is dominated by the heavy chain. The interface is centered on CDR-H3, with auxiliary contacts from CDR-H2; CDR-H1 contributes a single side-chain contact, whereas CDR-L2 is non-participatory. In the top-ranked model, the Omicron RBD epitope comprises the residues R346, A348, Y449, N450, K462, D467, I468, S469, E471, and G482. The corresponding antibody contacts arise predominantly from CDR-H3 positions R119, Y121, S122, N124, and Y128, supplemented by CDR-H2 (S50, T76, A77) and light-chain CDRs L1/L3 (Q84, S99). Across alternative acceptable docking solutions, the same CDR-H3 subset, R119, Y121, S122, N124, and Y128 recurred at the interface, indicating a conserved recognition motif for the Omicron variant (Table 1).

**TABLE 1:** Comparative interactions of XG83 with Wuhan and Omicron RBDs

Complex	Method	Docking scores	Antibody regions	RBD residues
XG83-Omicron RBD	Docking	(−280.18)	LC: Q84, S99 HC: S50, T76, A77, R119, Y121, S122, N124, Y128	R346, A348, Y449, N450, K462, D467, I468, S469, E471, G482

**Fig 2:**
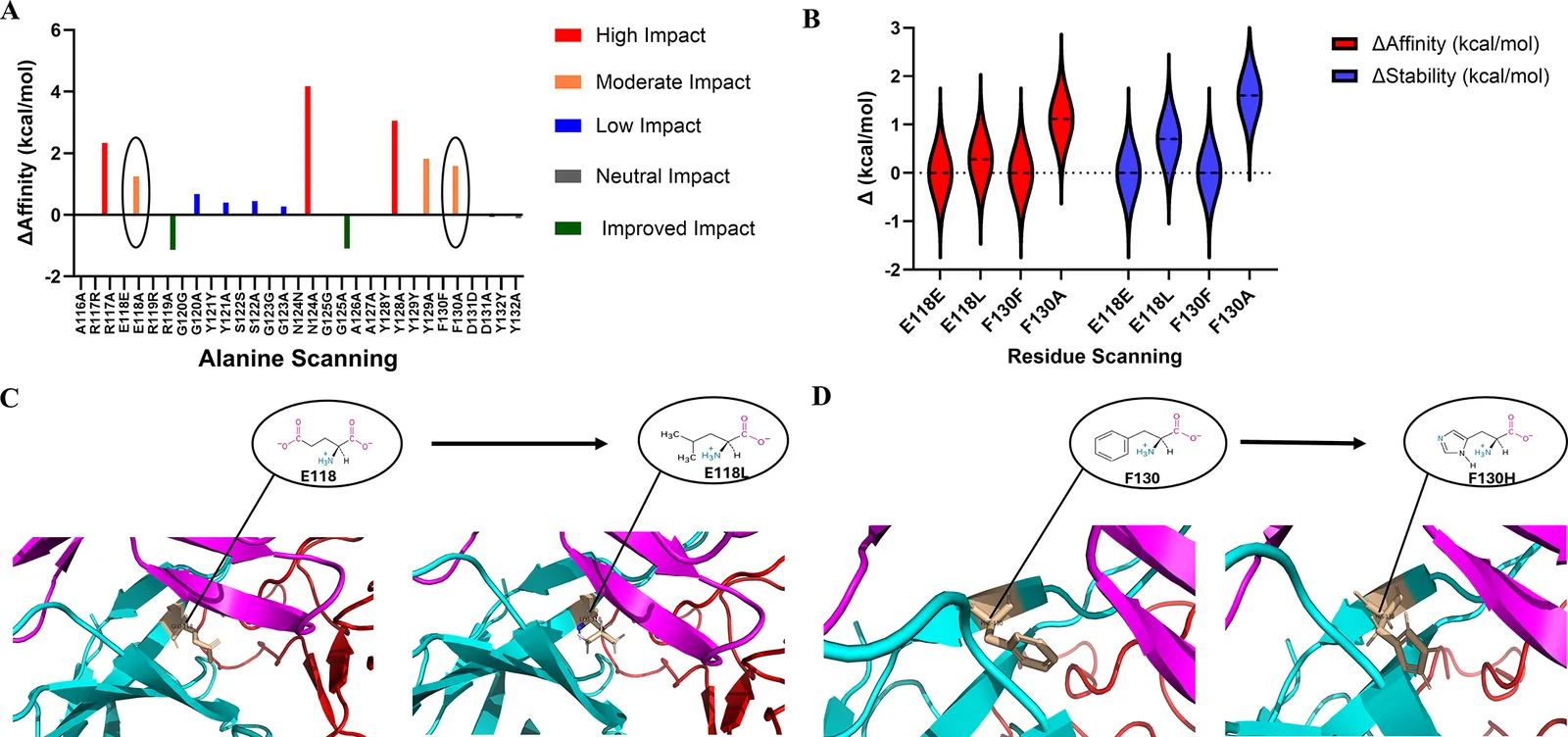
Alanine and residue scanning of XG83 CDR-H3 against Omicron RBD. (**A**) MOE alanine scan of CDR-H3 (A116–Y132). Bars show ΔAffinity (${Affinity}_{\text{mut}}$ - ${Affinity}_{\text{wt}}$, kcal·mol⁻¹); positive values indicate weaker binding, negative values improved binding. Color bins: high (red, ≥2.0), moderate (orange, 1.0–<2.0), low (blue, 0.1–<1.0), neutral (gray, |Δ|<0.1), improved (green, ≤−0.1). Hotspots: R117A, N124A, Y128A; moderate-impact sites: E118A, Y129A, F130A. (**B**) Residue scanning at E118 and F130. Violin plots show distributions of ΔAffinity (red) and ΔStability (blue) across sampled conformations; dashed lines mark medians. Positive values indicate reduced affinity/stability; negative values indicate improvements. (**C**) Model of E118L, where glutamate is replaced by leucine, removing charge and improving hydrophobic packing in the local pocket. (**D**) Model of F130H, an isosteric aromatic-to-polar swap that preserves ring size while introducing a polar face compatible with nearby residues.

MOE alanine scanning of XG83 CDR-H3 (A116-Y132) against the Omicron RBD (Fig. 2A) identified three primary hotspots whose mutation to alanine strongly reduced binding: N124A (+4.17 kcal·mol⁻¹), Y128A (+3.06 kcal·mol⁻¹), and R117A (+2.34 kcal·mol⁻¹). A second tier of residues showed moderate impact and, thus, warranted deeper analysis: E118A (+1.25 kcal·mol⁻¹), Y129A (+1.83 kcal·mol⁻¹), and F130A (+1.58 kcal·mol⁻¹). These positions likely report contributions from local electrostatics and hydrogen bonding (E118) as well as aromatic and hydrophobic packing within the CDR-H3 interface (Y129, F130). Most other substitutions were low-impact or functionally neutral (ΔAffinity ≤ ~0.5 kcal·mol⁻¹), whereas R119A (−1.13 kcal·mol⁻¹) and G125A (−1.10 kcal·mol⁻¹) modestly improved predicted affinity. MOE-reported ΔStability values for these mutations were small and did not alter residue prioritization (Table S2).

Guided by these data, we next carried out full residue-scanning at positions E118 and F130 to prioritize concrete substitutions for affinity optimization and to assess local steric compatibility (Fig. 2B through D). Y129, although identified as a moderate-impact site in the alanine scan, makes direct contacts with the RBD epitope and was, therefore, not pursued further to avoid disrupting the conserved recognition core. E118 and F130 were selected because their alanine variants exhibited both reduced affinity and positive ΔStability, suggesting that the wild-type chemistries at these positions may be suboptimal. At E118, alanine substitution led to weaker binding and predicted destabilization (E118A, ΔAffinity ≈ +1.25 kcal·mol⁻¹; ΔStability ≈ +1.60 kcal·mol⁻¹), implicating a delicate balance between charge, size, and packing at this site (Table S3). Residue scanning favored leucine as a conservative replacement that removes the unfavorable negative charge while preserving side-chain volume. The E118L model fits cleanly into the local pocket with no major clashes and forms tighter hydrophobic packing against neighboring framework residues (Fig. 2C), consistent with the energetic trends and highlighting E118 as an engineerable allosteric position.

For F130, alanine scanning likewise indicated a loss of affinity and structural destabilization upon the removal of the phenyl group (F130A, ΔAffinity ≈ +1.58 kcal·mol⁻¹; ΔStability ≈ +2.5 kcal·mol⁻¹) (Table S3). Residue scanning identified histidine as a near-isosteric substitute that preserves ring size while introducing tunable polarity. In the F130H model, the imidazole ring occupies the same pocket as the native phenylalanine without steric clashes (Fig. 2D), and its heteroatoms are oriented toward nearby polar groups, suggesting potential new hydrogen-bond or cation–π interactions. Taken together, the combined alanine- and residue-scanning workflow nominates E118L and F130H as structurally compatible, experiment-ready substitutions. These engineered positions lie outside the conserved CDR-H3 core that directly contacts the Omicron RBD, offering opportunities to modulate affinity and stability while preserving the fundamental epitope-recognition motif of XG83.

Overall, the combined alanine- and residue-scanning workflow E118L and F130H as structurally compatible, experiment-ready substitutions.

### Mutation docking and stability check

**Fig 3:**
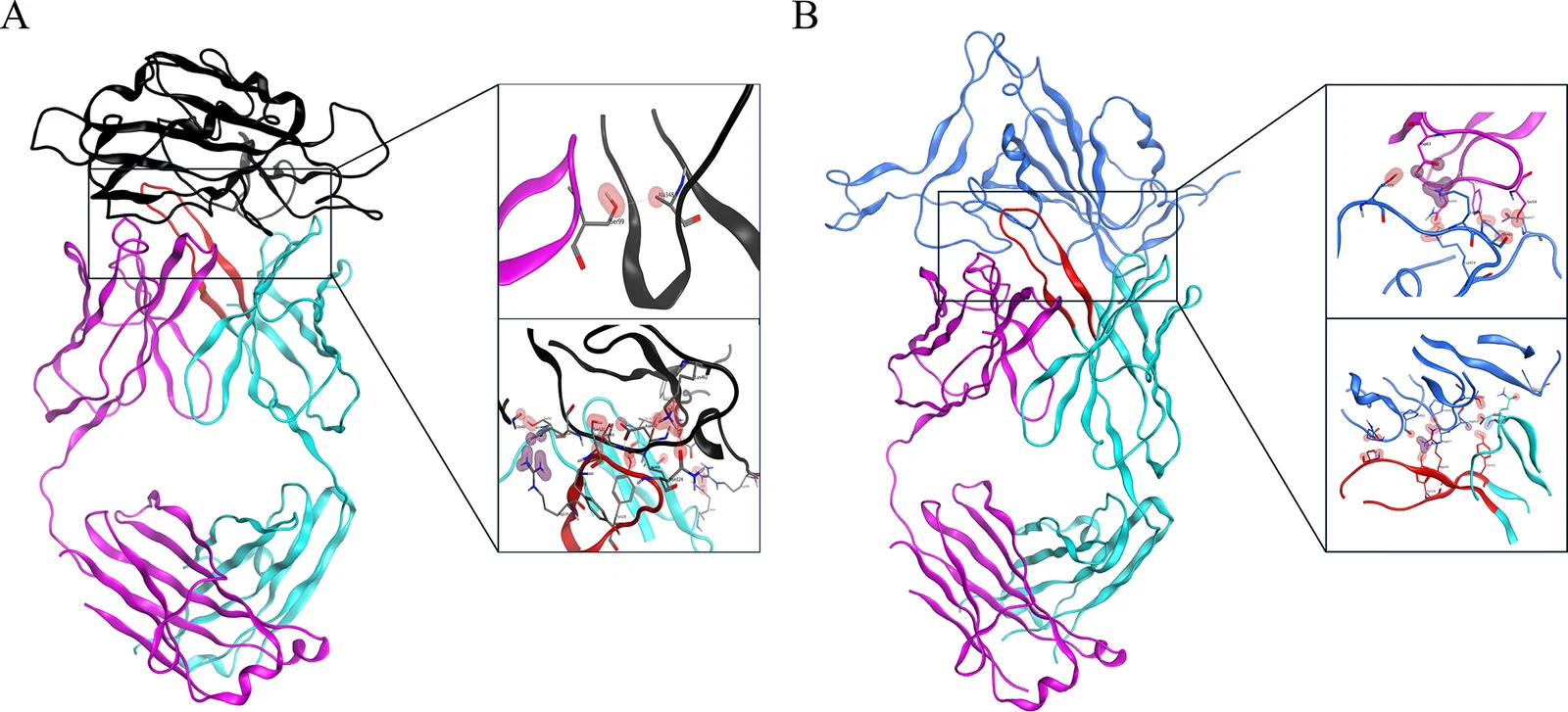
XG83 vs MuXG83 binding to Omicron RBD. (**A**) Parental XG83 engages Omicron RBD through a CDR-H3-centered interface; conserved loop residues E118, R119, Y121, S122, N124, Y128 contact an epitope anchored by N450 and I468 (Table 1). (**B**) Double-mutant MuXG83 (E118L/F130H) redistributes contacts re-engaging light-chain CDRs and expanding the epitope footprint to include 351–352 and 483/490/494 but yields a less favorable docking score (−254.2 vs −280.18; Table 2).

Docking of the parental XG83 Fab to the Omicron RBD recapitulates a CDR-H3-centered interface (Fig. 3A), in which a conserved subset of loop residues E118, R119, Y121, S122, N124, and Y128 recurrently contacts an epitope spanning R346, A348, Y449, N450, K462, D467, I468, S469, E471, and G482 (Table 2). The pose positions are R117/Y128 as primary anchors at the groove rim, with CDR-H2 providing supportive contacts and minimal contribution from CDR-L2. This arrangement yields the most favorable Omicron docking score for the parental antibody (−280.18; Table 2).

**Fig 4:**
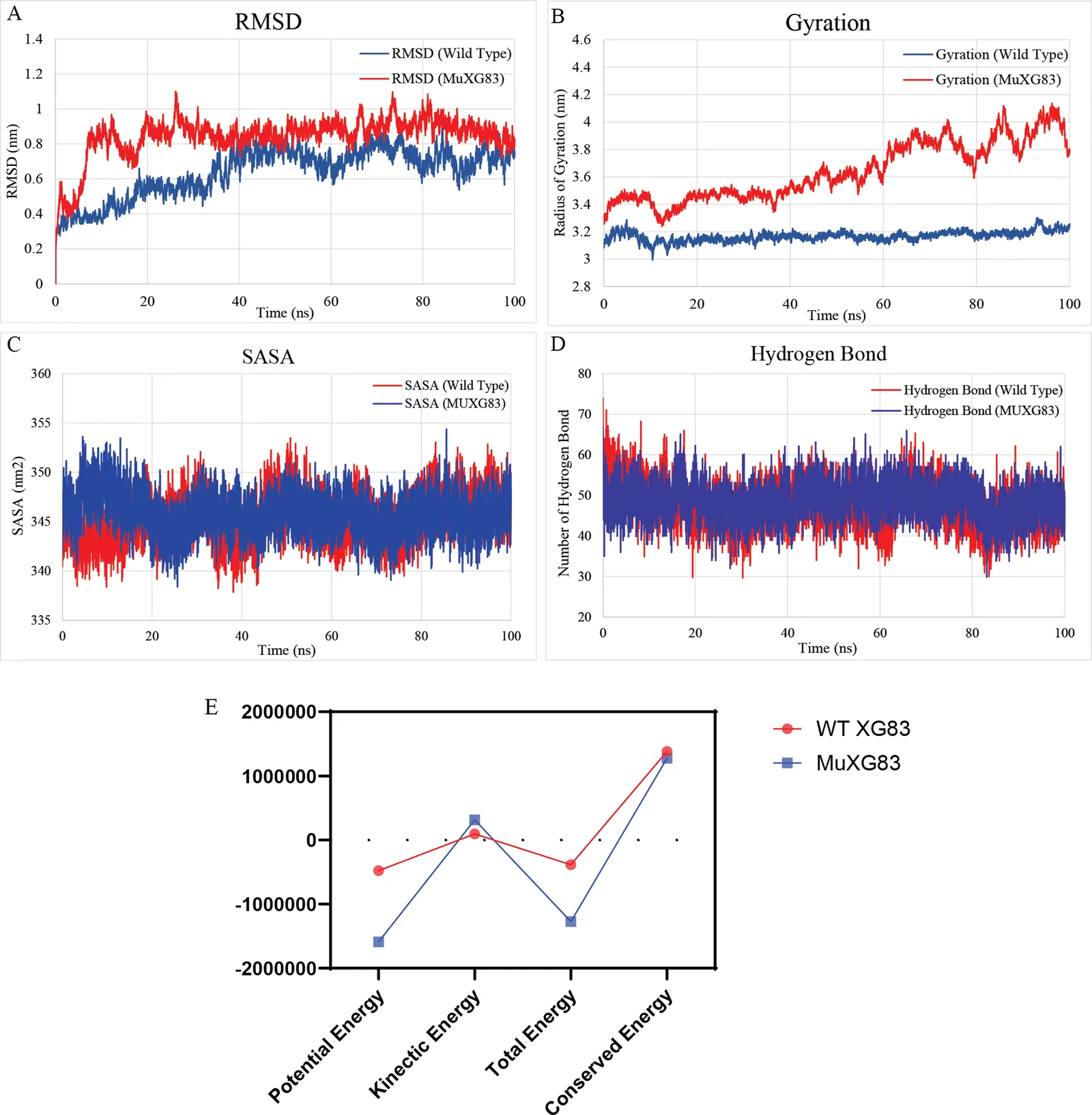
MD analysis of XG83 complexes with wild-type XG83-Omicron and MuXG83-Omicron RBDs. (**A**) RMSD (Cα-fitted) vs time shows the lowest drift for the wild-type XG83 complex with Omicron (red) and the highest for MuXG83-Omicron (blue). (**B**) Radius of gyration (Rg) indicates the wild-type XG83-Omicron complex is most compact; MuXG83-Omicron is more expanded and variable. (**C**) SASA of the complexes remains within a narrow range for the two systems, indicating no global unfolding. (**D**) Intermolecular hydrogen bonds at the Fab RBD interface fluctuate; Wuhan and MuXG83-Omicron tend to sustain higher counts than Omicron. (**E**) Average potential, kinetic, total, and conserved energy components extracted from the trajectories for wild-type XG83 (red circles) and MuXG83 (blue squares), illustrating broadly similar energetic profiles with modest differences between the two complexes.

**TABLE 2:** Mutation docking and interactions

Complex	Method	Docking scores	Antibody regions	RBD residues
MuXG83	Docking	−254.2	LC: Y34, S96, S99 HC: S50, T51, I71, L74, T76, N78, Q81, Q84, R119, Y121, Y128, Y129	346, 348, 351, 352, 446, 449, 450, 466, 468, 470, 471, 482, 483, 490, 494

In the engineered double mutant MuXG83 (E118L/F130H), the Omicron complex assumes a rebalanced geometry (Fig. 3B). The computed docking score is −254.2 (Table 2), less favorable than the parental model under the same scoring function, indicating no energetic gain. Despite this, the paratope redistributes heavy-chain contacts extend across S50, T51, I71, L74, T76, N78, Q81, Q84, R119, Y121, Y128, and T129, and the light chain is re-engaged through CDR-L1 (Y34) and CDR-L3 (S96, S99). On the epitope, the footprint broadens from 10 to 15 residues, adding contacts at 351–352 and 483/490/494 while retaining recurrent anchors at N450 and I468 (Tables 1 and 2). The mutant also samples a patch around 466/470/471 reminiscent of Wuhan-like engagement, consistent with a modest shift of the binding axis. In MuXG83, still the 118/130 don’t show any interactions, but the affinity has reduced.

To compare the conformational stability of WT XG83–Omicron RBD and MuXG83–Omicron RBD complexes, triplicate 100-ns molecular dynamics simulations were analyzed using the root mean square deviation (RMSD), radius of gyration (Rg), solvent-accessible surface area (SASA), and hydrogen-bond profiles. Average values were extracted at 1-ns intervals from each replicate trajectory and are summarized in Table 3. Across the simulations, the two complexes showed distinct convergence and stability patterns, with MuXG83 displaying higher structural deviation and reduced compactness compared with WT XG83.

**TABLE 3:** Triplicate MD simulation analysis of WT XG83-Omicron RBD and MuXG83-Omicron RBD complexes

Complex	Metric	Replicate 1 average	Replicate 2 average	Replicate 3 average	Final mean ± SD
WT XG83-Omicron RBD	RMSD (nm)	0.68	0.59	0.45	0.57 ± 0.12
WT XG83-Omicron RBD	Radius of gyration, Rg (nm)	3.15	3.10	3.12	3.12 ± 0.03
WT XG83-Omicron RBD	SASA (nm²)	348	346	349	347.67 ± 1.53
MuXG83-Omicron RBD	RMSD (nm)	0.85	0.81	0.82	0.83 ± 0.02
MuXG83-Omicron RBD	Radius of gyration, Rg (nm)	3.75	4.00	3.87	3.87 ± 0.13
MuXG83-Omicron RBD	SASA (nm²)	347	346	348	347.00 ± 1.00

The molecular dynamics (MD) simulations revealed clear stability differences between the wild-type XG83 Omicron RBD complex and its double-mutant counterpart, MuXG83 (E118L/F130H). In the RMSD profile (Fig. 4A), the wild-type complex (red) equilibrated rapidly within the first 20 ns and maintained an average RMSD of ~0.55 nm, indicating structural convergence and minimal deviation from the initial docked conformation. In contrast, the mutant complex (blue) displayed persistent fluctuations, stabilizing only after 70 ns at an elevated RMSD of ~0.85–1.0 nm, reflecting greater backbone flexibility and reduced conformational stability. Consistent with this, the radius of gyration (RG) analysis (Fig. 4B) showed a more compact and stable architecture for the wild-type complex (average RG ≈ 3.25 nm) compared to the mutant (~3.6–3.9 nm). The gradual expansion of RG in the MuXG83 trajectory suggests that mutations at E118 and F130 disrupt optimal packing within the Fab-RBD interface, increasing the dynamic looseness of the binding region.

Surface area analyses (Fig. 4C) revealed that solvent-accessible surface area (SASA) remained lower and more consistent in the wild-type complex (mean ≈ 345 nm²) than in MuXG83 (mean ≈ 350–355 nm²). The elevated SASA in the mutant corresponds to increased solvent exposure of hydrophobic residues at the interface consistent with a weakened and more open binding conformation. To improve the statistical robustness of the MD analysis, three independent 100-ns simulations were performed for both WT XG83–Omicron RBD and MuXG83–Omicron RBD complexes. RMSD, radius of gyration, and SASA values were extracted at 1-ns intervals from each trajectory, and replicate averages were used to calculate the final mean ± SD values (Table 3). Across the three simulations, WT XG83–Omicron RBD showed a lower average RMSD of 0.57 ± 0.12 nm, whereas MuXG83–Omicron RBD showed a higher RMSD of 0.83 ± 0.02 nm, indicating greater structural deviation in the mutant complex. Similarly, the radius of gyration was lower for WT XG83–Omicron RBD (3.12 ± 0.03 nm) than for MuXG83–Omicron RBD (3.87 ± 0.13 nm), suggesting that the mutant complex adopted a less compact conformation. In contrast, SASA values were comparable between WT and mutant complexes, with 347.67 ± 1.53 nm² for WT and 347.00 ± 1.00 nm² for MuXG83, indicating no major global unfolding. Overall, the triplicate MD analysis supports that the E118L/F130H substitutions increase conformational deviation and reduce compactness of the XG83–Omicron RBD complex (Table 3).

Hydrogen-bond analysis (Fig. 4D) further confirmed these findings. The wild-type complex maintained an average of 50–55 intermolecular hydrogen bonds, with brief spikes up to 70, indicating robust and dynamic hydrogen-bonding stability. In contrast, the MuXG83 complex exhibited a lower and more fluctuating hydrogen-bond count (typically 40–45), highlighting transient contact patterns and reduced persistence of interfacial interactions. This weakened hydrogen-bond network likely contributes to the reduced docking score observed for MuXG83.

Energy profiling of the Omicron complexes indicates that the wild-type XG83-Omicron complex is energetically more stable than the MuXG83-Omicron mutant. In the bar analysis, the wild type exhibits more favorable (more negative) potential energy and more negative total energy than MuXG83, consistent with a tighter, better-packed interface; the mutant shows less favorable (less negative) potential/total terms and only modest differences in kinetic and “conserved” energy components. Taken together, the energy decomposition aligns with our MD/stability readouts and supports that the wild-type XG83 complex forms a thermodynamically superior interaction with the Omicron RBD, whereas MuXG83 is comparatively weakened (Fig. 4E).

The replicate-based MD analysis showed that WT XG83-Omicron RBD maintained lower RMSD and Rg values than MuXG83-Omicron RBD, indicating greater conformational stability and compactness. In contrast, SASA values were comparable between the two complexes, suggesting that the E118L/F130H mutations did not cause global unfolding but promoted a less compact and more dynamically variable complex

### PCA and covariance analysis

**Fig 5:**
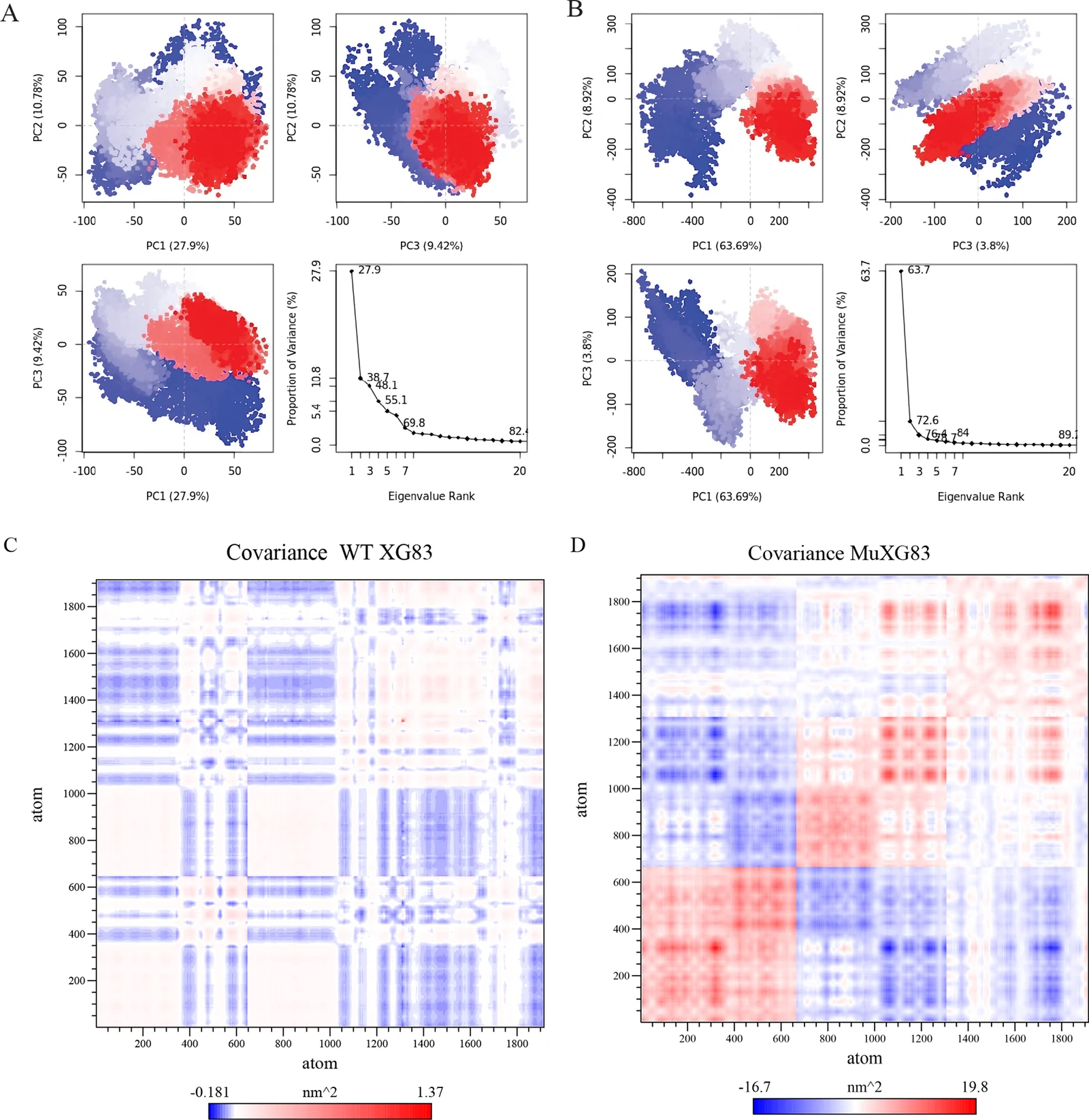
Principal-component analysis (PCA) of conformational dynamics and covariance of atomic displacements of the WT XG83 and MuXG83. (**A**) PCA of wild-type XG83-Omicron MD: projections on PC1–PC2, PC2–PC3, and PC1–PC3 with variance explained noted; points shaded light→dark from early→late simulation, showing a single, compact basin. Scree plot highlights a gradual variance decay (PC1 = 27.9%). (**B**) PCA of MuXG83-Omicron MD: projections reveal broader, split basins and greater spread, dominated by PC1 (63.69%). Scree plot exhibits a sharp elbow, indicating one prevailing collective motion. Together, the mutant samples a larger, less constrained conformational space than the wild type. (**C**) Wild-type XG83-Omicron complex: predominantly weak off-diagonal covariance with a narrow dynamic range (−0.18 to 1.37 nm²), consistent with restrained, locally coordinated motions. (**D**) MuXG83-Omicron complex: strong block-level positive (red) and negative (blue) covariances over a much broader range (−16.7 to 19.8 nm²), indicating larger-amplitude, long-range coupled motions and reduced dynamical stability.

Principal component analysis (PCA) of the MD trajectories shows that the wild-type XG83-Omicron complex explores a compact conformational basin with modest dispersion across. Wild-type (Fig. 5A) motion is compact and largely isotropic across the first three PCs. PC1, PC2, and PC3 explain 27.9%, 10.78%, and 9.42% of the variance (cumulative m, 8.1%). Projections onto PC1–PC2, PC2–PC3, and PC1–PC3 show a single dense cloud with high overlap between early and late frames (light to dark shading), indicating rapid equilibration to a single metastable basin with minor local breathing. The scree plot decays gradually (no dominant mode), consistent with distributed, low-amplitude fluctuations. This pattern matches the lower RMSD, tighter radius of gyration, higher H-bond persistence, and slightly lower SASA observed for the wild type.

MuXG83 mutant (Fig. 5B), dynamics are dominated by a single collective coordinate: PC1 captures 63.69% of the variance (PC2 = 8.92%, PC3 = 3.80%, cumulative 76.4%). The PC1–PC2 and PC1–PC3 projections are elongated and partially bimodal, with late frames migrating along PC1 toward a second cluster, indicating slow transitions between sub-states rather than confinement to one basin. The broader, anisotropic spread suggests large-amplitude rocking/twisting motions of the Fab relative to the RBD and enhanced flexibility of the CDR-H3 tip and adjacent CDR-H2/L3 segments.

The atomic covariance maps reveal markedly different dynamical coupling patterns between the two complexes. In the wild-type XG83-Omicron map (Fig. 5C), most off-diagonal elements are weak with alternating small blue/red streaks, indicating modest, locally coordinated motions and limited long-range coupling between domains. The color scale range is narrow (≈−0.18 to 1.37 nm²), consistent with restrained fluctuations and a well-packed interface. In contrast, the MuXG83 (E118L/F130H)-Omicron map (Fig. 5D) exhibits large, block-level positive and negative covariances spanning entire subdomains (strong red/blue quadrants), reflecting enhanced interdomain coupling and anti-correlated motions. The magnitude range expands dramatically (≈−16.7 to 19.8 nm²), indicating larger-amplitude collective motions. Prominent cross-blocks suggest decoupling between the Fab variable domains and the RBD and increased internal coupling within the antibody loops features that align with the PCA/RMSD/Rg findings and support a model in which the E118L/F130H substitutions disrupt CDR-H3 pre-organization, loosen the interface, and permit long-range rocking/twisting motions absent in the wild type.

### Experimental validation of XG83 mutation

**Fig 6:**
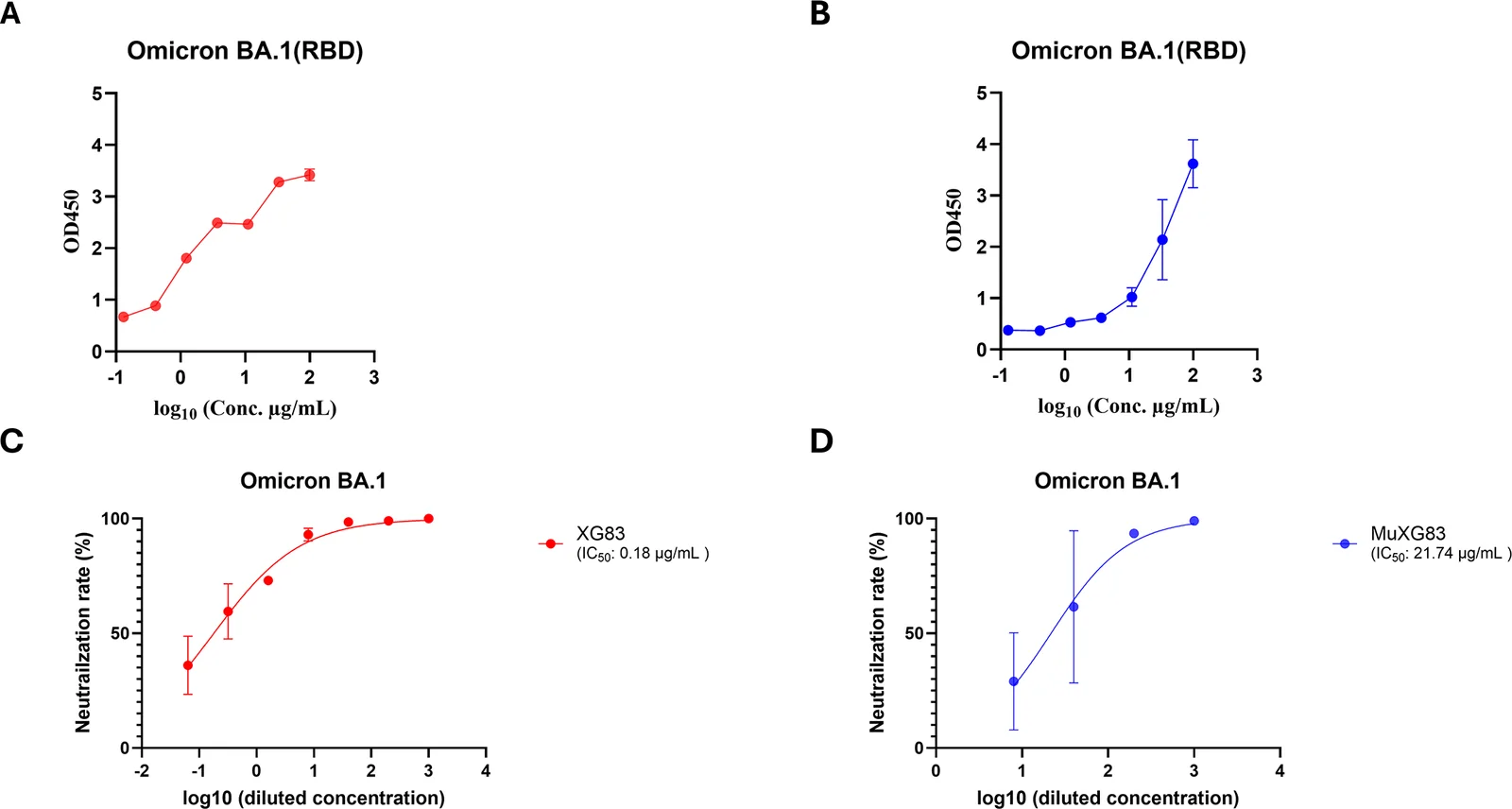
Evaluation of the binding activities and neutralization of Omicron BA.1 by XG83 and MuXG83. (**A**) High antibody concentrations result in robust binding, as seen by the concentration-dependent rise in OD450 in the wild-type XG83 ELISA-binding curve, shown red in color. (**B**) MuXG83 (blue) ELISA-binding curve, showing much lower peak OD450 and delayed response compared to wild type, as well as substantially decreased binding over the same dose range. Data are presented as mean ± SD from two technical replicates (*n* = 2) and plotted on a log10 concentration scale. (**C**) The XG83 neutralization curve in red color shows its effectiveness in neutralizing the Omicron BA.1 strain with IC_50_ value. (**D**) The capacitance of Omicron BA.1 to MuXG83 with its neutralization curve is shown in blue color. Curves show neutralization rate (%) vs log₁₀ (diluted antibody concentration); error bars and neutralization values represent mean ± SD of duplicates of nonlinear regression (curve fit). GraphPad Prism 10.4.1 has been used to generate the plots.

ELISA binding to Omicron BA.1 RBD showed that wild-type XG83 bound more strongly than the E118L/F130H double-mutant MuXG83 (Fig. 6A and B). Across the titration range, WT XG83 produced consistently higher OD450 values than MuXG83 at nearly all matched concentrations. Overall, WT XG83 showed approximately 40% higher apparent binding, with up to a 1.7-fold difference compared with MuXG83 (Fig. 6A and B). Only at the highest concentration tested, 2 µg/mL did MuXG83 reach an OD450 value comparable to WT XG83, suggesting that the mutant may retain similar maximal binding capacity under saturating conditions but has reduced apparent binding potency (Fig. 6A and B). These findings indicate that simultaneous E118L and F130H substitutions weaken, rather than enhance, antigen recognition.

Consistent with the ELISA trend, pseudovirus neutralization assays showed a much larger functional reduction for MuXG83 (Fig. 6C and D). WT XG83 neutralized Omicron BA.1 more efficiently across the tested antibody concentrations, with an IC₅₀ of approximately 0.18 µg/mL (Fig. 6C). In contrast, MuXG83 showed markedly reduced neutralizing activity, with an IC₅₀ of approximately 21.74 µg/mL, corresponding to an approximately 120-fold loss of potency relative to WT XG83 (Fig. 6D). Thus, the E118L/F130H mutations reduced both apparent RBD binding and neutralization efficiency against Omicron BA.1 (Fig. 6A through D).

### SPR analysis

**Fig 7:**
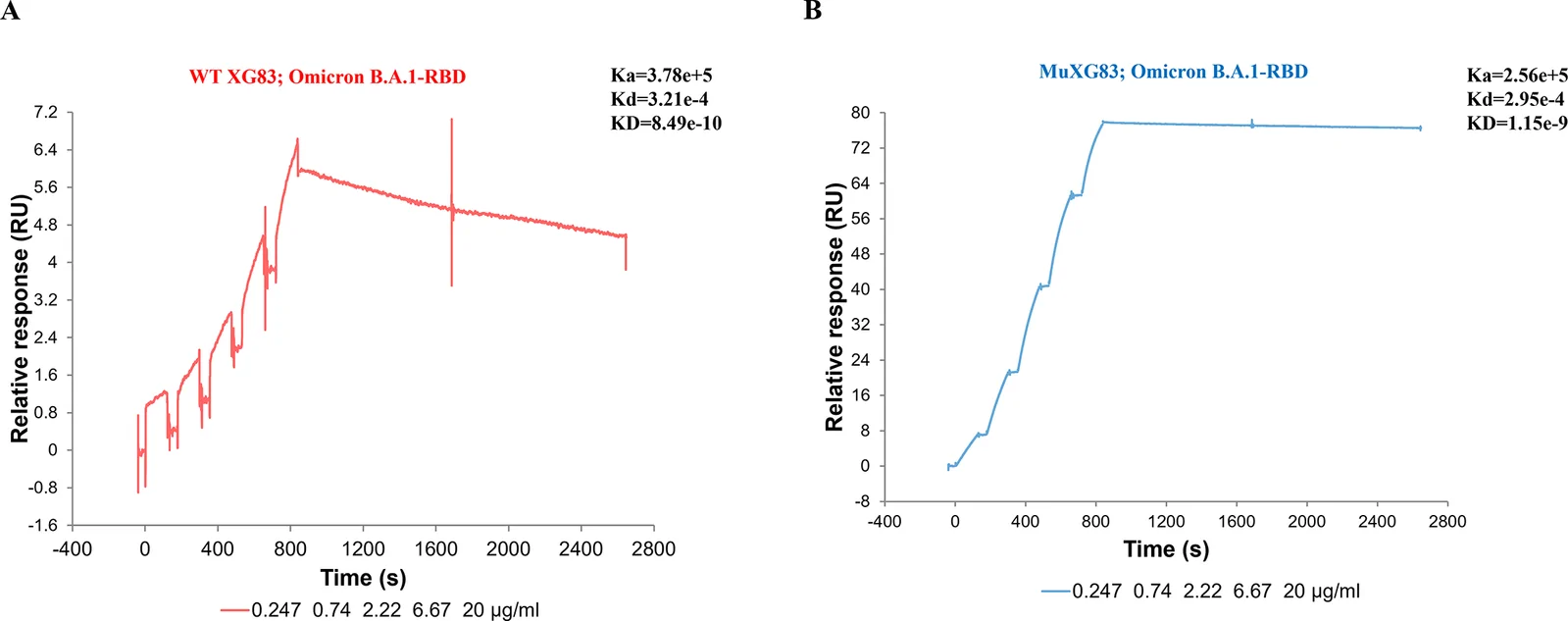
Sensor grams of surface plasmon resonance (SPR) depicting the binding of WT XG83 and MuXG83 to the Omicron BA.1 receptor-binding domain (RBD). Representative SPR sensor grams illustrating the real-time interaction patterns of wild-type XG83 and the E118L/F130H mutant (MuXG83) with the Omicron BA.1 receptor-binding domain (RBD). The *x*-axis denotes time (S), while the *y*-axis signifies response units (RU). The ascending segment of each sensor gram represents the association phase, whereas the descending segment indicates dissociation after analyte administration. WT XG83 had superior overall binding reactions in comparison to MuXG83. Sensorgrams are shown with distinct RU scales for enhanced visualization clarity.

To determine whether the ELISA signal reflected true binding affinity, SPR analysis was performed for WT XG83 and MuXG83 binding to Omicron BA.1 RBD. Consistent with the ELISA findings, WT XG83 displayed stronger binding affinity than MuXG83. WT XG83 showed a KD of 8.49 × 10⁻¹⁰ M, whereas MuXG83 showed a KD of 1.15 × 10⁻⁹ M, corresponding to an approximately 1.35-fold reduction in affinity for the mutant (Fig. 7).

Kinetic analysis showed that WT XG83 had a higher association rate than MuXG83, with Ka values of 3.78 × 10⁵ and 2.56 × 10⁵ 1/Ms, respectively. The dissociation rates were relatively similar between WT XG83 and MuXG83, suggesting that the E118L/F130H mutations mainly reduce the efficiency of antigen association rather than causing a major increase in dissociation. Together, the SPR data confirm that WT XG83 has stronger affinity for Omicron BA.1 RBD and support the conclusion that E118L/F130H weakens antigen recognition.

## DISCUSSION

SARS-CoV-2 continues to diversify antigenically, challenging first-generation monoclonal antibodies and motivating designs that target more conserved features of the spike RBD. In this context, XG83 is compelling because its paratope dominated by CDR-H3 engages a relatively conserved patch, offering a starting point for breadth-oriented engineering (17–20). The study asked whether structure-guided mutation analysis (alanine scanning and residue scanning) could identify tractable substitutions that improve binding to Omicron while preserving the conserved recognition mode. Methodologically, the combined crystallography of XG83 bound to Wuhan RBD with comparative modeling and docking to Omicron, followed by 100-ns MD simulations to assess complex stability. *In silico* alanine scanning prioritized energetically important paratope residues, residue scanning nominated clash-free substitutions, and an engineered double mutant (MuXG83: E118L/F130H) were tested by ELISA, SPR, and pseudo-virus neutralization against Omicron RBD.

The key results are threefold. First, contact mapping showed Wuhan engages more-and-more balanced CDR contacts than Omicron (Table 1), with conserved anchors at N450 and I468. Second, alanine scanning highlighted R117 and Y128 as hot spots (ΔAffinity ≥ +2 kcal·mol⁻¹), while E118, Y129, and F130 appeared engineerable (ΔAffinity ≤ −1 kcal·mol⁻¹; Table S1); residue scanning triaged E118L and F130H for follow-up (Fig. 6; Table S1). Third, although the MuXG83-Omicron docking score (−254.2) was less favorable than parental XG83-Omicron (−280.18), the mutant broadened its epitope footprint and restored light-chain engagement; MD indicated higher flexibility than Wuhan but partial recovery of interfacial H-bonding relative to Omicron. The E118L/F130H substitutions should, therefore, be interpreted cautiously. Although these residues lie outside the main conserved CDR-H3 contact core, MuXG83 showed weaker ELISA binding and markedly reduced pseudovirus neutralization compared with WT XG83 (Fig. 6A through D). In the absence of compensatory rescue mutations or a dedicated long-range energetic network analysis, these findings do not prove a defined allosteric mechanism. Rather, they suggest that non-contact CDR-H3 substitutions can indirectly disturb the binding-competent conformation of XG83, likely by altering local packing, loop stability, and Fab–RBD interface organization. These results show that the E118L/F130H substitutions reduce apparent binding and neutralization potency against Omicron BA.1. The study observations align with prior work on RBD-directed antibodies, which distinguishes RBM-focused class 1/2 nAbs often exquisitely potent yet mutation-sensitive from class 3/4 nAbs that bind more conserved scaffolds and better tolerate antigenic drift. The predominance of CDR-H3 in recognition and the role of second shell/allosteric positions are well documented in these systems and motivate our alanine-scanning strategy to separate direct hot spots from residues whose replacement can relieve suboptimal electrostatics or sterics. The CDR-H3 scanning strategy initially aimed to identify mutations that could improve XG83 activity against Omicron BA.1 RBD. However, functional validation showed that the E118L/F130H substitutions weakened binding and substantially reduced neutralization. This opposite outcome suggests that the XG83 CDR-H3 region is structurally constrained and has limited tolerance for mutation. Thus, the main value of the scanning workflow was not the identification of an improved mutant, but the discovery of fragile CDR-H3 positions where apparently tolerated substitutions can disrupt antibody function. Strengths of this study include an integrated pipeline that links a solved Fab-RBD crystal structure to docking, MD, and energetic scanning and residue-level prioritization that produced concrete, clash-free designs (E118L, F130H) rather than generic improve-the-interface proposals. Also, the cross-validation of computational predictions with experimental ELISA and neutralization; and a comparative analysis across Wuhan, Omicron, and a rationally engineered variant, revealing which interactions are conserved vs remodelable. Head-to-head comparisons of XG83 and MuXG83, plus combination studies with non-overlapping epitope partners, will clarify additivity/synergy and barrier-to-escape. A limitation of this study is that experimental validation was performed only against Omicron BA.1. Therefore, the present data cannot establish expanded breadth or a general breadth–potency trade-off across SARS-CoV-2 variants. Testing XG83 and MuXG83 against a broader variant panel, including Wuhan, Delta, BA.2, BA.5, XBB, and other antigenically divergent RBDs, will be necessary to determine whether the altered contact footprint observed computationally translates into any measurable breadth advantage. A further limitation is that neutralization was evaluated using an Omicron BA.1 pseudo virus system rather than authentic live SARS-CoV-2. Although pseudovirus assays are widely used for comparative neutralization screening, they may not fully reproduce all features of live-virus infection. Therefore, future studies should validate WT XG83 and MuXG83 neutralization using authentic SARS-CoV-2 variants under appropriate biosafety conditions.

Parallel assessments of developability (FcRn binding, thermostability, polyreactive) will inform lead selection. The study design objective is a breadth-preserving, Omicron-competent derivative of XG83 that targets a conserved RBD patch with sufficient affinity/kinetic stability to neutralize circulating variants and provide a foundation for a combination product. The structural rules derived here-hot-spot conservation, light-chain re-engagement, and avoidance of steric penalties at CDR-H3-offer actionable guidance for subsequent cycles of affinity maturation and Fc engineering. Structure-guided mutation analysis of XG83 identified engineerable paratope positions that improve stability to Omicron without abandoning conserved epitope recognition; MuXG83 is a first, experimentally validated step in that direction. Extending these insights to neutralization and breadth will determine its suitability as a therapeutic lead.

### Conclusion

An integrated, structure-guided workflow anchored by the XG83-Wuhan RBD crystal structure and augmented with computational alanine/residue scanning, docking, and molecular dynamics identified E118 and F130 as engineerable CDRH3 positions with potential allosteric leverage on binding. Alanine scanning showed increased ΔStability at these sites (e.g., E118A, F130A), consistent with the idea that side-chain remodeling can reweight local packing and long-range dynamics; conversely, residues predicted to be destabilizing exhibited decreased stability upon substitution. In conclusion, this study shows that structure-guided CDR-H3 engineering of XG83 can identify non-epitope residues with allosteric influence on antibody–RBD recognition. However, the experimentally tested E118L/F130H mutant reduced binding and neutralization against Omicron BA.1 compared with parental XG83. These findings indicate that structurally compatible CDR-H3 substitutions may still destabilize the functional antibody–RBD complex and impair potency. Broader variant-panel testing will be required before conclusions regarding neutralization breadth or breadth–potency trade-offs can be made. Overall, the E118L/F130H mutant should be interpreted as a negative design outcome, demonstrating that computationally nominated CDR-H3 substitutions may reduce rather than improve antibody function. This finding identifies structural constraints within the XG83 CDR-H3 loop and emphasizes the need for experimental validation before claiming affinity optimization or breadth enhancement.

## Data Availability

All data sets generated and analyzed during this study are available upon request.

## SUPPLEMENTAL MATERIAL

Supplemental tables spectrum.04127-25-s0001.docx Tables S1 and S2.

ASM does not own the copyrights to Supplemental Material that may be linked to, or accessed through, an article. The authors have granted ASM a non-exclusive, world-wide license to publish the Supplemental Material files. Please contact the corresponding author directly for reuse.

## References

[1] Bordoloi D, Xu Z, Ho M, Purwar M, Bhojnagarwala P, Cassel J, Giron LB, Walker S, Kulkarni AJ, Ruiz ET, et al.. 2021. Identification of novel neutralizing monoclonal antibodies against SARS-CoV-2 spike glycoprotein. ACS Pharmacol Transl Sci 4:1349–1361. doi:10.1021/acsptsci.1c00058 34396059 PMC8353887

[2] Yang H, Rao Z. 2021. Structural biology of SARS-CoV-2 and implications for therapeutic development. Nat Rev Microbiol 19:685–700. doi:10.1038/s41579-021-00630-8 34535791 PMC8447893

[3] Rexhepaj M, Asarnow D, Perruzza L, Park Y-J, Guarino B, Mccallum M, Culap K, Saliba C, Leoni G, Balmelli A, et al.. 2024. Isolation and escape mapping of broadly neutralizing antibodies against emerging delta-coronaviruses. Immunity 57:2914–2927. doi:10.1016/j.immuni.2024.10.001 39488210 PMC12279085

[4] Cofas-Vargas LF, Mendoza-Espinosa P, Montalvo-Sandoval FD, Pérez-Rodríguez S, Rauda-Ceja JA, Hernández-Peralta P, Durán-Vargas A, Trujillo-Roldán MA, Valdez-Cruz NA, García-Hernández E. 2025. A unified topology-based classification of SARS-CoV-2 RBD neutralizing antibodies systematizes affinity trends across variants. MAbs 17:2575083. doi:10.1080/19420862.2025.2575083 41117360 PMC12542604

[5] Li W, Chen Y, Prévost J, Ullah I, Lu M, Gong SY, Tauzin A, Gasser R, Vézina D, Anand SP, et al.. 2022. Structural basis and mode of action for two broadly neutralizing antibodies against SARS-CoV-2 emerging variants of concern. Cell Rep 38:110210. doi:10.1016/j.celrep.2021.110210 34971573 PMC8673750

[6] Alshahrani M, Parikh V, Foley B, Raisinghani N, Verkhivker G. 2025. Quantitative characterization and prediction of the binding determinants and immune escape hotspots for groups of broadly neutralizing antibodies against omicron variants: atomistic modeling of the SARS-CoV-2 spike complexes with antibodies. Biomolecules 15:249. doi:10.3390/biom15020249 40001552 PMC11853647

[7] Xie J, Ding C, He J, Zhang Y, Ni S, Zhang X, Chen Q, Wang J, Huang L, He H, et al.. 2021. Novel monoclonal antibodies and recombined antibodies against variant SARS-CoV-2. Front Immunol 12:715464. doi:10.3389/fimmu.2021.715464 34539645 PMC8442604

[8] Haider S, Ahmad N, Shafiq M, Siddiqui AR, Nur-E-Alam M, Ahmed A, Ul-Haq Z. 2025. Uncovering the binding mechanism of mutated omicron variants via computational strategies. ACS Omega 10:2790–2798. doi:10.1021/acsomega.4c08562 39895721 PMC11780447

[9] Rath SL, Padhi AK, Mandal N. 2022. Scanning the RBD-ACE2 molecular interactions in Omicron variant. Biochem Biophys Res Commun 592:18–23. doi:10.1016/j.bbrc.2022.01.006 35007846 PMC8732900

[10] Stein SC, Ssebyatika G, Benecke T, Ströh L, Rajak MK, Vollmer B, Menz S, Waldmann J-Y, Tipp SN, Ochulor O, et al.. 2025. A critical residue in a conserved RBD epitope determines neutralization breadth of pan-sarbecovirus antibodies with recurring YYDRxxG motifs. MBio 16:e00606–25. doi:10.1128/mbio.00606-25 40742150 PMC12421856

[11] Wang J, Ni S, Chen Q, Wang C, Liu H, Huang L, Nasir MW, Wang W, Zhang X, Wu J, et al.. 2024. Discovery of a novel public antibody lineage correlated with inactivated SARS‐CoV‐2 vaccine and the resultant neutralization activity. J Med Virol 96:e70073. doi:10.1002/jmv.70073 39610345

[12] Wang Y, Zhang Z, Yang M, Xiong X, Yan Q, Cao L, Wei P, Zhang Y, Zhang L, Lv K, et al.. 2024. Identification of a broad sarbecovirus neutralizing antibody targeting a conserved epitope on the receptor-binding domain. Cell Rep 43:113653. doi:10.1016/j.celrep.2023.113653 38175758

[13] Cabrera A, Hernández LH, Chávez D, Medina-Franco JL. 2018. Molecular modeling of potential dual inhibitors of HIV reverse transcriptase and integrase. CMB 08:1–41. doi:10.4236/cmb.2018.81001

[14] Vanommeslaeghe K, Hatcher E, Acharya C, Kundu S, Zhong S, Shim J, Darian E, Guvench O, Lopes P, Vorobyov I, et al.. 2010. CHARMM general force field: a force field for drug-like molecules compatible with the CHARMM all-atom additive biological force fields. J Comput Chem 31:671–690. doi:10.1002/jcc.21367 19575467 PMC2888302

[15] Huang J, MacKerell AD. 2013. CHARMM36 all-atom additive protein force field: validation based on comparison to NMR data. J Comput Chem 34:2135–2145. doi:10.1002/jcc.23354 23832629 PMC3800559

[16] Abraham MJ, Murtola T, Schulz R, Páll S, Smith JC, Hess B, Lindahl E. 2015. GROMACS: High performance molecular simulations through multi-level parallelism from laptops to supercomputers. SoftwareX 1–2:19–25. doi:10.1016/j.softx.2015.06.001

[17] Harris C, Kapingidza AB, San JE, Christopher J, Gavitt T, Rhodes B, Janowska K, O’Donnell C, Lindenberger J, Huang X, et al.. 2025. Design of SARS-CoV-2 RBD immunogens to focus immune responses towards conserved coronavirus epitopes. bioRxiv. doi:10.1101/2025.01.09.632180 PMC12282168 40511920

[18] Feng H, Wang Z, Li L, Li Y, Lu M, Chen X, Hu L, Sun Y, Du R, Qin R, et al.. 2025. Discovery of synergistic broadly neutralizing antibodies targeting non-dominant epitopes on SARS-CoV-2 RBD and NTD. Vaccines (Basel) 13:592. doi:10.3390/vaccines13060592 40573924 PMC12197520

[19] Wu W-L, Chiang C-Y, Lai S-C, Yu C-Y, Huang Y-L, Liao H-C, Liao C-L, Chen H-W, Liu S-J. 2022. Monoclonal antibody targeting the conserved region of the SARS-CoV-2 spike protein to overcome viral variants. JCI Insight 7:e157597. doi:10.1172/jci.insight.157597 35290246 PMC9089791

[20] Starr TN, Czudnochowski N, Liu Z, Zatta F, Park Y-J, Addetia A, Pinto D, Beltramello M, Hernandez P, Greaney AJ, et al.. 2021. SARS-CoV-2 RBD antibodies that maximize breadth and resistance to escape. Nature 597:97–102. doi:10.1038/s41586-021-03807-6 34261126 PMC9282883

