# Supplementary material for: Allosteric effects of CDR-H3 mutations modulate binding and neutralization of a conserved SARS-CoV-2 RBD-targeting antibody
Source: Microbiol Spectr. 2026 Aug 5;14(9):e04127-25. doi: 10.1128/spectrum.04127-25 (PMC13532266; doi:10.1128/spectrum.04127-25)
Supplement: Supplementary file 1 [file spectrum.04127-25-s0001.docx]

**Table S1: Summary of X-ray crystallographic data quality and validation statistics for the xg83 antibody in complex with the WT Wuhan SARS-CoV-2 RBD.**

| **Crystallographic Parameters** | **Obtain Values with reference** |
| --- | --- |
| Ramachandran Favored | 96.42% |
| Ramachandran Outliers | 0 % |
| Clash score | 6.35 |
| Completeness | 95.63 (97.89) (%) Reference value= over 90% |
| CC1/2 (Correlation Coefficient) | 0.995 (0.775) Reference value= 1 |
| R-meas | 0.2209 (1.769) Reference value=below 3.10 Å |
| Protein residues | 627 |
| Resolution Range | 2.95 Å Reference value=below 3.10 Å |
| MolProbity score | 2.13 Reference value=below 2.20Å |

**Table S2: MOE-based alanine scanning analysis of the XG83–Omicron RBD complex, showing the effects of single-point mutations on binding affinity and complex stability.**

| **Complex** | **Mutation** | **Affinity** | **dAffinity** | **Stability** | **dStability** |
| --- | --- | --- | --- | --- | --- |
| XG83-Omicron complex | A116A | -32.3825 | 0 | 938366.7 | 0 |
| XG83-Omicron complex | R117R | -36.6996 | 0 | 938376.4 | 0 |
| XG83-Omicron complex | R117A | -34.3647 | 2.335 | 938378 | 1.5522 |
| XG83-Omicron complex | E118E | -58.8537 | 0 | 937717.8 | 0 |
| XG83-Omicron complex | E118A | -57.6063 | 1.2474 | 937719.4 | 1.609 |
| XG83-Omicron complex | R119R | -50.8005 | 0 | 937173.416 | 0 |
| XG83-Omicron complex | R119A | -51.9307 | -1.1302 | 937175.514 | 2.0983 |
| XG83-Omicron complex | G120G | -61.1624 | 0 | 937716.415 | 0 |
| XG83-Omicron complex | G120A | -60.4826 | 0.6798 | 937716.454 | 0.0393 |
| XG83-Omicron complex | Y121Y | -50.1619 | 0 | 937163.273 | 0 |
| XG83-Omicron complex | Y121A | -49.7648 | 0.3971 | 937164.001 | 0.7279 |
| XG83-Omicron complex | S122S | -46.0901 | 0 | 937718.957 | 0 |
| XG83-Omicron complex | S122A | -45.6460 | 0.4440 | 937719.711 | 0.7546 |
| XG83-Omicron complex | G123G | -47.1535 | 0 | 937719.059 | 0 |
| XG83-Omicron complex | G123A | -46.8808 | 0.2727 | 937719.173 | 0.1143 |
| XG83-Omicron complex | N124N | -47.4353 | 0 | 937725.387 | 0 |
| XG83-Omicron complex | N124A | -43.2635 | 4.1718 | 937726.000 | 0.6130 |
| XG83-Omicron complex | G125G | -41.4217 | 0 | 937815.101 | 0 |
| XG83-Omicron complex | G125A | -42.5214 | -1.0998 | 937815.201 | 0.1003 |
| XG83-Omicron complex | A126A | -55.9488 | 0 | -55.9488 | 0 |
| XG83-Omicron complex | A127A | -46.5567 | 0 | 937812.049 | 0 |
| XG83-Omicron complex | Y128Y | -66.9273 | 0 | 937158.182 | 0 |
| XG83-Omicron complex | Y128A | -63.8690 | 3.0583 | 937159.735 | 1.5530 |
| XG83-Omicron complex | Y129Y | -50.5379 | 0 | 937811.230 | 0 |
| XG83-Omicron complex | Y129A | -48.7117 | 1.8262 | 937813.740 | 2.5099 |
| XG83-Omicron complex | F130F | -40.8127 | 0 | 937811.156 | 0 |
| XG83-Omicron complex | F130A | 937811.156 | 1.5835 | 937813.629 | 2.4733 |
| XG83-Omicron complex | D131D | -35.7800 | 0 | 937821.090 | 0 |
| XG83-Omicron complex | D131A | -35.8431 | -0.0631 | 937821.592 | 0.5017 |
| XG83-Omicron complex | Y132Y | -31.8802 | 0 | 938366.158 | 0 |
| XG83-Omicron complex | Y132A | -31.8696 | 0.0106 | 938367.032 | 0.8740 |

Table S3: Residues Scanning and structure impact

| **Complex** | **Mutation** | **Affinity** | **dAffinity** | **Stability** | **dStability** |
| --- | --- | --- | --- | --- | --- |
| XG83-Omicron complex | E118E | -58.4977 | 0 | 937717.8 | 0 |
| XG83-Omicron complex | E118L | -58.2174 | 0.2802 | 937718.5 | 0.7 |
| XG83-Omicron complex | F130F | -40.9095 | 0 | 937811 | 0 |
| XG83-Omicron complex | F130H | -39.7953 | 1.1142 | 937812.6 | 1.6 |
